# Supplementary material for: Honey bulk DNA metagenomic analysis to identify honey biological composition and monitor honey bee pathogens
Source: NPJ Sci Food. 2025 May 30;9:91. doi: 10.1038/s41538-025-00464-1 (PMC12125302; doi:10.1038/s41538-025-00464-1)
Supplement: Supplementary file 1 — Supplementary Information [file 41538_2025_464_MOESM1_ESM.pdf]

Honey bulk DNA metagenomic analysis to identify honey biological composition and monitor honey bee pathogens

Priit Paluoja, Mihkel Vaher, Hindrek Teder, Kaarel Krjutškov, Andres Salumets, Kairi Raime

Supplementary Information

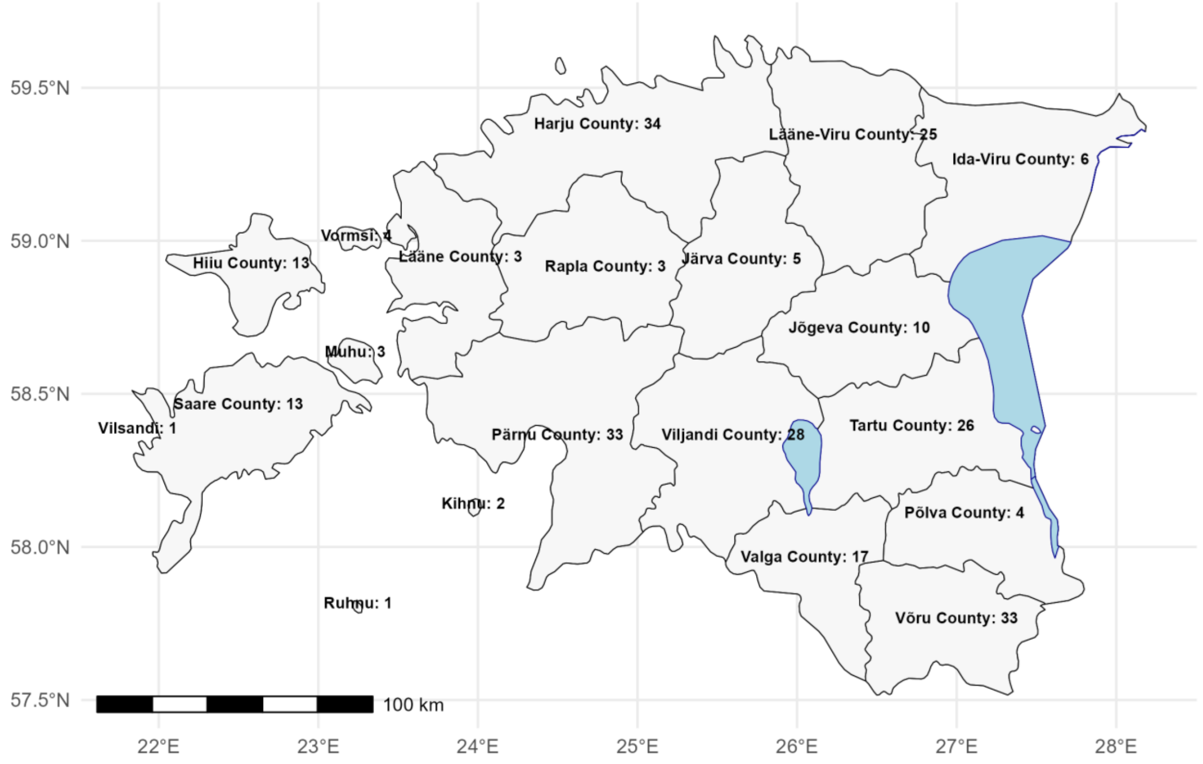

S1 Fig. Geographical distribution of 264 Estonian honey samples used in the study.

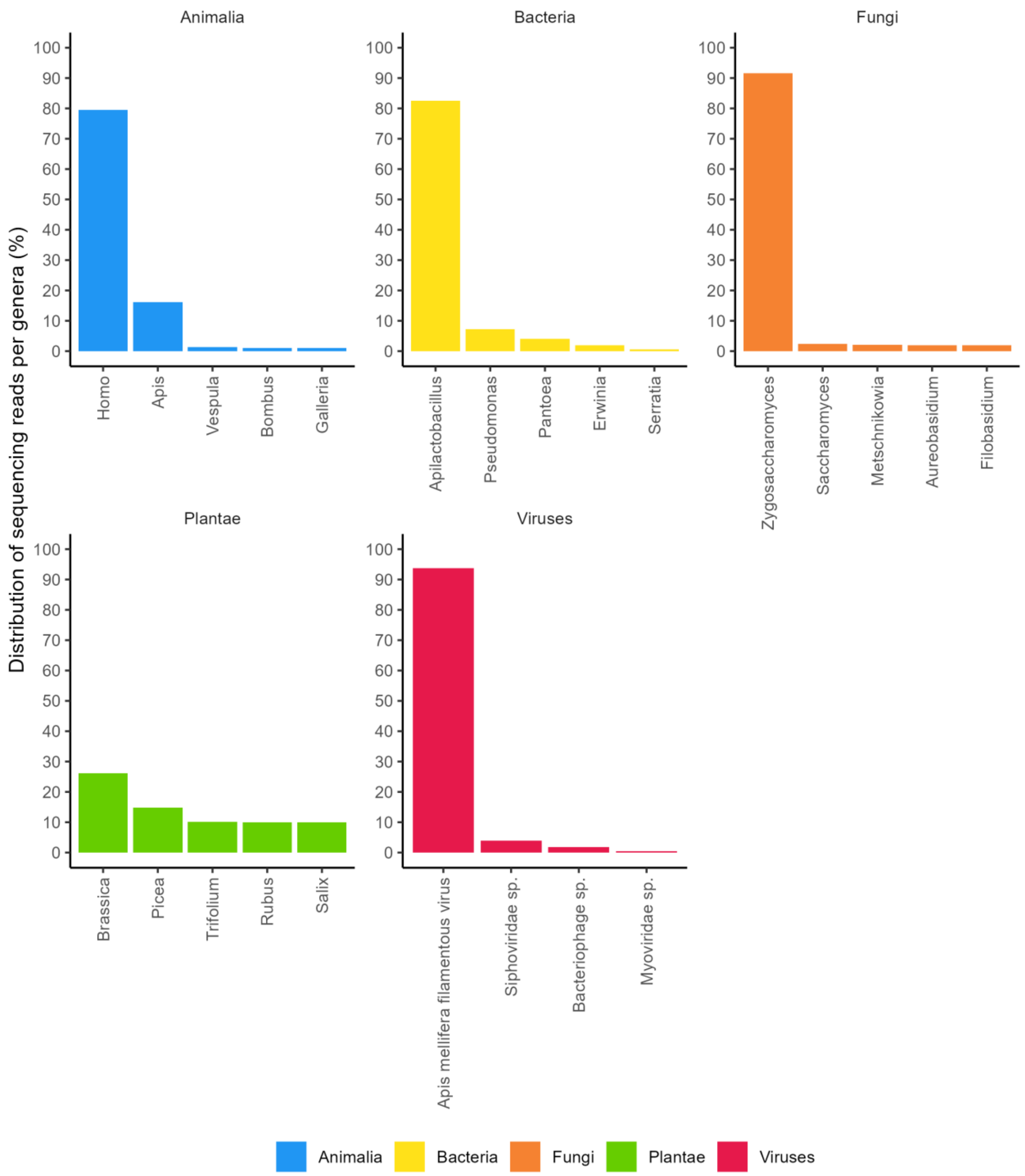

S2 Fig. Common genera of Bacteria, Fungi, Animalia, Plantae, and Viruses from the DNA of Estonian honey.

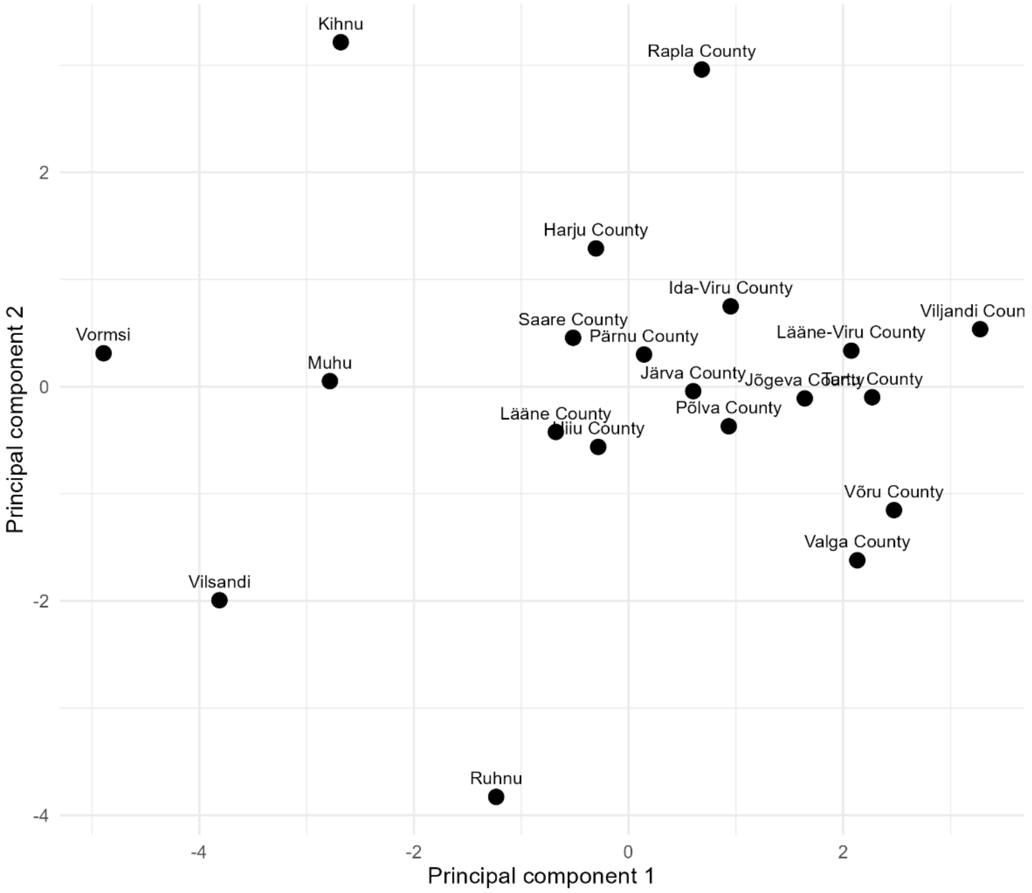

S3 Fig. Principal component analysis (PCA) of species profiles by Estonian geographical region. The analysis shows that the islands (Vormsi, Vilsandi, Muhu, Kihnu) generally differ from the mainland, with the exception of Saare County, which is also an island. Additionally, Rapla County shows distinct differences, but overall, islands differ from the mainland in their species profiles.

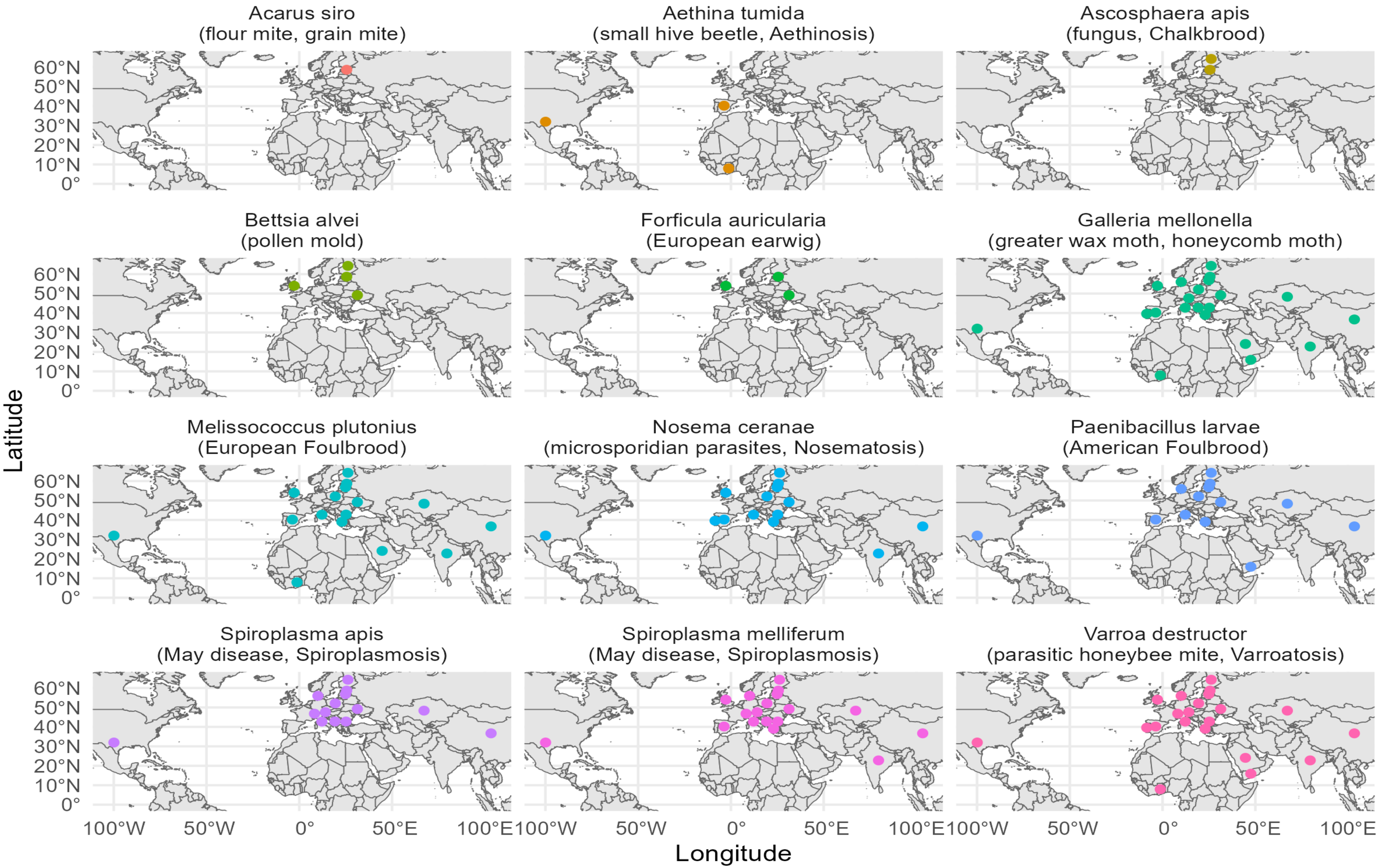

S4 Fig. Global distribution of pathogen detection by country. We analyzed 369 samples, including 266 from Estonia. 130 data points are shown, as some foreign honey was a mix of blends and not included. Out of 20 targeted pathogens, 12 were detected.

Table S1. Origins of 103 foreign honey samples utilized in the pathogen analysis.

Origin	N
Austria	1
Bulgaria	9
China	10
Denmark	2
England	2
Finland	3
Germany	1
Ghana	1
Greece (including Rhodes Island)	4
India	4
Italy	1
Kazakhstan	1
Latvia	4
Mix of EU and non-EU honey	18
Montenegro	1
Non-EU honey (including Ukraine, Central and South American honey)	7
Poland	3
Portugal	1
Saudi Arabia	1
Scotland	3
Spain	2
Switzerland	2
Ukraine	17
United Arab Emirates	1
Unspecified EU honey	2
USA	1
Yemen	1
